# Arthroscopic surgical treatment of complex femoroacetabular impingement with massive labral calcification and gracilis autograft reconstruction: a case report and literature review

**DOI:** 10.3389/fsurg.2026.1770189

**Published:** 2026-06-11

**Authors:** Gang Li, Feifei Shen, Xingtao Ge, Wei Sun

**Affiliations:** 1 Department of Orthopedics, Qilu Hospital, Cheeloo College of Medicine, Shandong University, Qingdao, China; 2 Department of Pathology, Qilu Hospital, Cheeloo College of Medicine, Shandong University, Qingdao, China

**Keywords:** arthroscopy, femoroacetabular impingement, gracilis autograft, labral calcification, labral reconstruction

## Abstract

This study reports a rare case of femoroacetabular impingement (FAI) combined with massive acetabular labrum calcification. The patient was a 48-year-old female sports enthusiast who had experienced right hip pain for over six months. Despite conservative treatment aimed at reducing swelling and alleviating pain, she still suffered from hip pain and restricted movement. She underwent right hip arthroscopy under general anesthesia, including removal of the calcified lesion of the acetabular labrum, reconstruction of the labrum with autologous gracilis tendon, and femoral head-neck reshaping. At the nine-month follow-up, the patient's hip function had improved significantly, with no complications such as nerve or vascular injury, wound infection, or heterotopic ossification. This case suggests that hip arthroscopy with labral reconstruction may represent a viable treatment option that may contribute to improved symptoms and function in patients with FAI combined with large labral calcifications; it may facilitate return to sports and enhance quality of life, potentially offering a therapeutic strategy for the clinical management of such conditions.

## Introduction

Femoroacetabular Impingement (FAI) is a prevalent condition affecting the hip joint. It is primarily characterized by hip pain and limited mobility, which significantly compromises patients' quality of life. Recently, advancements in sports medicine have substantially refined the clinical understanding of FAI. FAI morphology is classified into cam, pincer, and mixed types. The overall prevalence of FAI morphology ranges from 10% to 74% (1). The diagnosis of FAI requires comprehensive clinical judgment, integrating characteristic symptoms, signs, and radiological evidence of impingement morphology, rather than relying solely on imaging abnormalities (2, 3). FAI is considered the primary cause of hip pain in adolescents and young adults (4). A 2026 prospective national study in Japan, conducted among patients at specialized hip clinics, reported a primary FAI prevalence of 6.2% among those presenting with groin pain (5). In athletic populations, the prevalence of FAI is generally higher. For instance, the prevalence of symptomatic FAI was 42% among French professional table tennis players (6), whereas the overall prevalence reached 85.6% among Brazilian professional soccer players (7).

Acetabular labral calcification, a degenerative condition that often results in hip pain and functional limitations, is relatively uncommon in the context of FAI but should not be overlooked (8, 9). The concomitant presence of FAI and acetabular labral calcification complicates both diagnosis and treatment. Currently, there is no consensus among clinicians regarding the optimal management strategies for such cases. According to epidemiological surveys, the prevalence of acetabular ossification variations is about 3.33% in the general population, rising to 8.65% in symptomatic FAI patients (10). Acetabular ossification variations can be classified into several types, such as the labral calcification, acetabular ossicles, and acetabular rim stress fractures, among which the labral calcification is the most common type (11–13). Epidemiological studies estimate that acetabular labral calcification occurs in approximately 1.83% of the general population, with most cases involving small, asymptomatic calcifications (10). Furthermore, Schmitz CC et al. noted that labral calcifications associated with FAI typically range from 1.6 to 5.4 mm in diameter, and around 20% of these cases present multiple calcifications (14, 15). Xiaodong Ju et al. reported that the diameter of labral calcifications in FAI cases ranged from 9.5 to 13.2 mm, and all these cases present single lesions (16). However, the case we reported is FAI with a notably large calcification measuring approximately 40 mm × 20 mm × 5 mm.

Management of femoroacetabular impingement with concomitant labral calcification includes both conservative and surgical modalities. Initial management consists of conservative measures, including pharmacotherapy, physical therapy, and activity modification. Surgical intervention is reserved for patients with severe structural lesions, persistent symptoms, or for those whose conditions are refractory to conservative management. Compared with traditional open surgery, hip arthroscopy offers many advantages including direct intra-articular visualization, limited tissue trauma, enhanced surgical precision, shorter recovery time, etc (8, 9, 17, 18). The patient in the present case is a 48-year-old female sports enthusiast who had been experiencing right hip pain for more than six months. After the failure of standardized conservative management, the patient underwent arthroscopic femoroplasty, debridement of the calcified labral lesion, and labral reconstruction with an autologous gracilis tendon graft. Follow-up assessments conducted at 9 months postoperatively demonstrated significant improvement in hip function. This case report suggests that arthroscopic debridement with labral reconstruction maybe a feasible treatment for FAI with substantial labral calcification. This surgical technique may lead not only to improvements in symptoms and function but also to an increased likelihood of return to sporting activities, thereby improving overall quality of life while offering new clinical perspectives for the clinical management of this condition.

## Case report

A 48-year-old female sports enthusiast presented with right hip pain and mild limitation of movement, that began approximately six months prior in the absence of obvious trauma. The symptoms significantly worsened over the following two months. The hip pain was persistent, and the symptoms worsened after prolonged sitting and walking, but could be slightly relieved after rest. Despite conservative treatments, including oral nonsteroidal anti-inflammatory drugs and physiotherapy, being ineffective, she presented to the department of sports medicine at our institution on July 13, 2025. After physical examination and further evaluation via magnetic resonance imaging (MRI) and radiography of the right hip, she was diagnosed with right femoroacetabular impingement syndrome and right acetabular labrum calcification, and was subsequently admitted to our hospital for surgical treatment.

**Figure 1:**
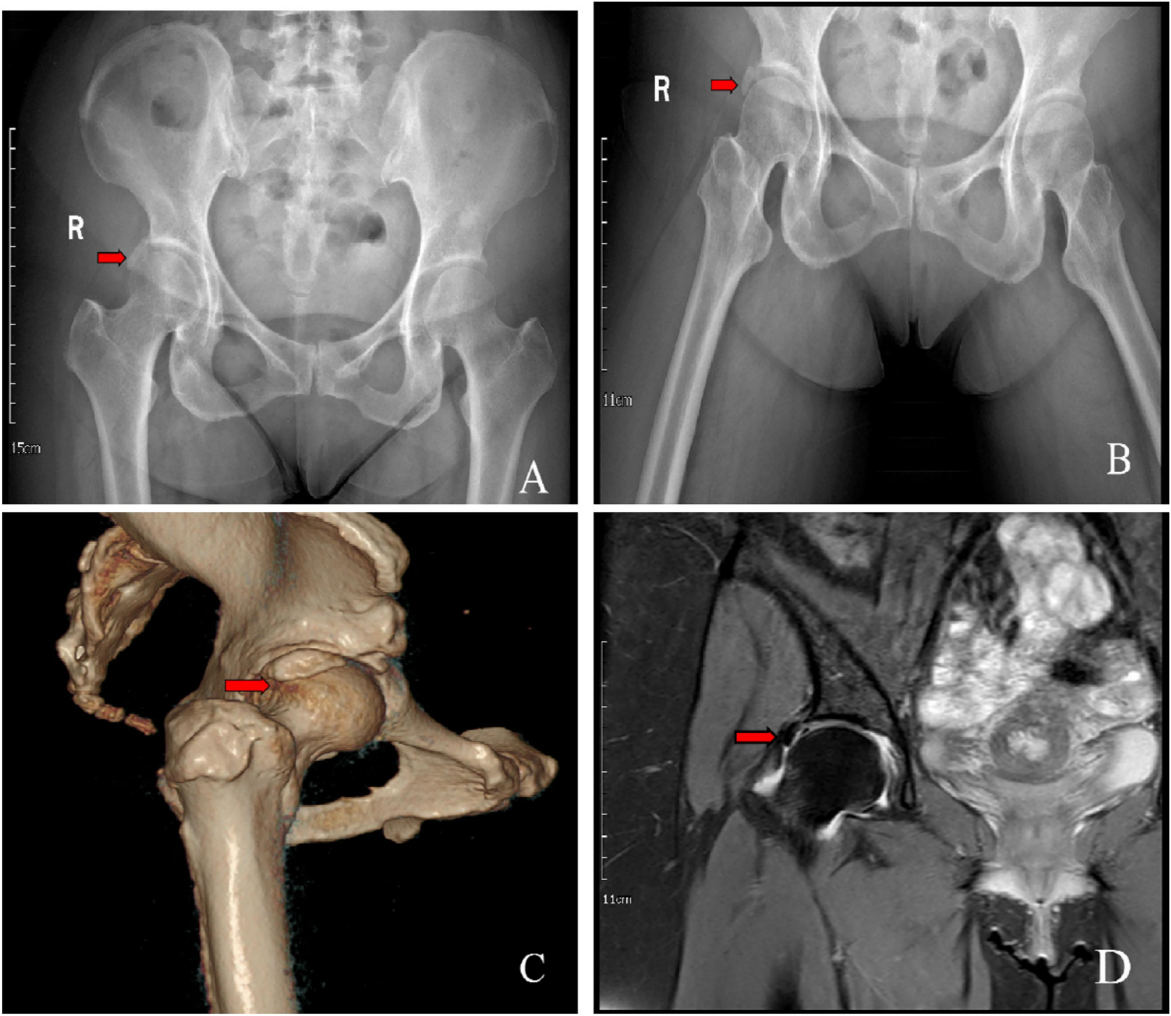
Preoperative radiographs [**(A)** anteroposterior view; **(B)** dunn view] and three-dimensional CT [**(C)** reconstructed view] demonstrate a high-density shadow at the anterolateral edge of the right acetabulum. Preoperative MRI [**(D)** Coronal view] shows right acetabular labrum calcification and injury.

The laboratory tests, electrocardiogram (ECG), and bilateral lower extremity vascular ultrasound revealed no significant abnormalities. The anteroposterior pelvic radiograph and the right hip Dunn view radiograph demonstrated calcification of the right acetabular labrum, with a right neck-shaft angle of 132°, a lateral center-edge (LCE) angle of 28°, a Tönnis angle of 9°, a Sharp angle of 37°, an alpha angle of 70°, and no obvious crossover sign (Figures 1A, B). A three-dimensional computed tomography (CT) scan of the right hip showed a calcified lesion at the anterior edge of the right acetabulum, with a maximum diameter of approximately 4 cm (Figure 1C). Magnetic resonance imaging (MRI) of the right hip revealed calcification of the acetabular labrum, along with joint effusion (Figure 1D). Lidocaine (6–8 mL at 1%) was administered for a diagnostic hip injection.

Physical examination showed no obvious swelling in the right hip joint; tenderness was noted in the inguinal and greater trochanter regions. Significant limitation of right hip movement: Flexion 100° (left hip 130°), Abduction 40° (left hip 60°), Internal rotation 30° (left hip 40°), External rotation 30° (left hip 50°). The flexion adduction internal rotation (FADIR) test, flexion abduction external rotation (FABER) test, and Patrick's test were all positive. The roll-over test and straight leg raise test were negative. The Beighton score was 0; the preoperative VAS score was 7; the Modified Harris Hip Score (mHHS) was 60; and the Non-Arthritic Hip Score (NAHS) was 50.

Her diagnosis was right hip FAI (Cam type) and right acetabular labrum calcification. She was informed of the efficacy, risks, and alternative options for surgical treatments. The patient agreed to the treatment plan and signed the surgical consent form.

### Surgical technique

#### Anesthesia and position

**Figure 2:**
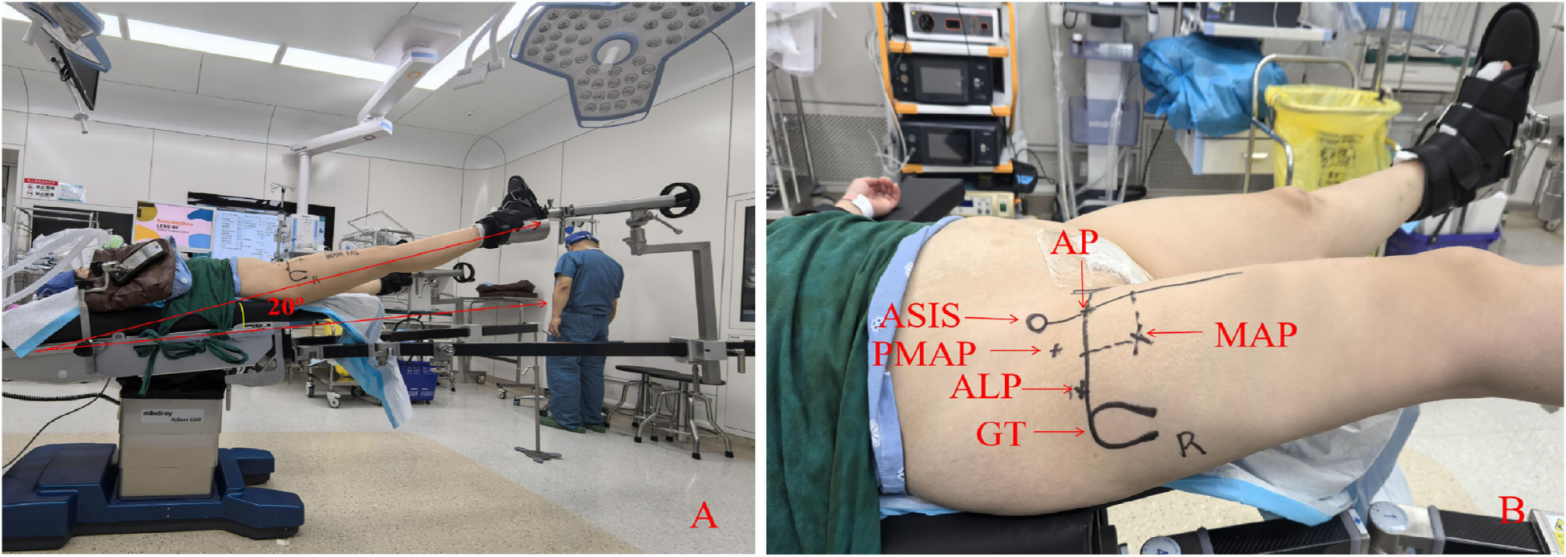
Patient's position and surgical portals. **(A)** The patient was placed in the supine position on a hip arthroscopy-specific traction bed without a perineal post, with traction applied to the right lower limb. **(B)**: The surgical portals illustrated are the anterolateral portal (ALP), mid-anterior portal (MAP), proximal mid-anterior portal (PMAP), and anterior portal (AP).

During surgery, the patient was placed in the supine position on a hip arthroscopy-specific traction bed without a perineal post, with traction applied to the right lower limb. The patient was positioned in a Trendelenburg position with an angle of approximately 20°. An abdominal binder was also applied to increase the friction between the patient and the bed surface, facilitating adequate hip joint distraction (Figure 2A).

#### Arthroscopic portals

After general anesthesia, the anterolateral portal (ALP), mid-anterior portal (MAP), and proximal mid-anterior portal (PMAP) were established (Figure 2B). The conventional ALP was used as the viewing portal. The MAP and PMAP were used as working portals. The outside-in technique was employed to resect the soft tissue anterior to the hip joint capsule, exposing the capsule and creating a T-shaped incision to enter the right hip joint cavity.

#### Diagnostic examination

**Figure 3:**
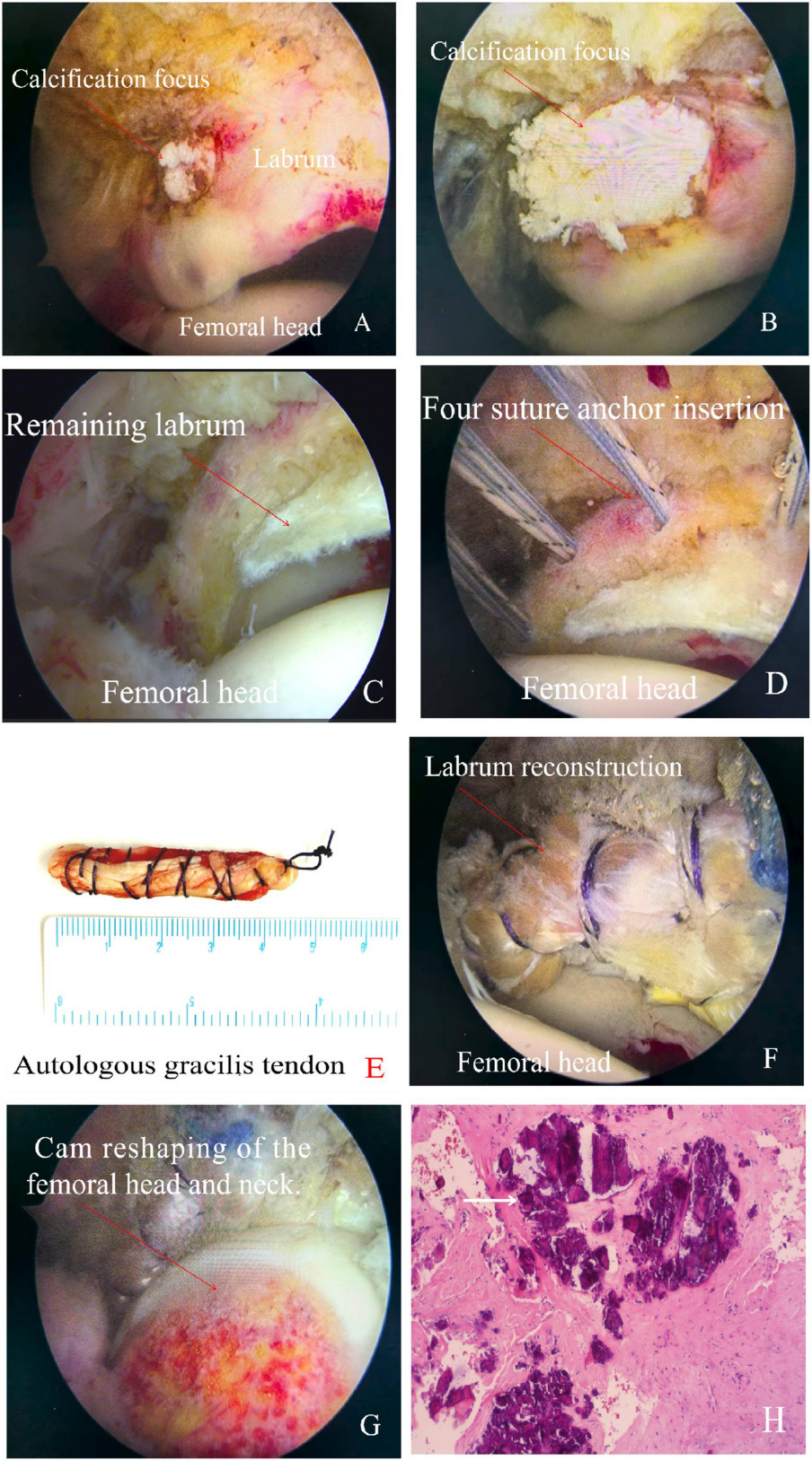
Arthroscopic findings and pathology results. **(A)** The labrum is congested and swollen, with a calcification focus in the labrum, as indicated by the red arrow; **(B)** The white toothpaste-like calcification extrudes from the labrum upon compression with a probe hook, as indicated by the red arrow. **(C)** Complete debridement of the calcific focus. **(D)** Four absorbable suture anchors were placed at the outer edge of the acetabulum. **(E)** Harvesting of the autologous gracilis tendon. **(F)** Labral reconstruction using the autologous gracilis tendon. **(G)** Cam reshaping of the femoral head and neck. **(H)** Pathology results reveal abundant calcified tissue and scant fibrous tissue, with the white arrow indicating the calcified component.

Under traction of the right lower extremity, the central compartment of the hip joint was explored. The articular cartilage of the hip joint was classified as Tönnis grade 0. The labrum between the 11:00 and 14:00 positions exhibited a severe inflammatory reaction, appearing erythematous, hypertrophic, and edematous. Mechanical debridement of the labrum using a shaver revealed extensive white, toothpaste-like calcific deposits within the tissue (Figures 3A, B).

#### Calcification removal and treatment of labrum

Under arthroscopic guidance, the shaver was used to remove the calcific deposits. After thoroughly removing the calcific deposits, the remaining labrum was of very poor quality, with a labrum defect of about 4 cm in length and less 2 mm in width (Figure 3C). Traction was reapplied to the affected limb. The acetabular rim was freshened with a burr, and four 3.0 mm absorbable suture anchors were placed at 11:00, 12:00, 1:00, and 2:00 at 2 mm from the outer edge of the acetabulum (Figure 3D). It was decided to harvest the autologous gracilis tendon to perform labral reconstruction. The traction on the lower limb was released; the knee was flexed to 30°and a 3-cm incision was made on the medial side of the tibial tuberosity to expose the gracilis tendon. The gracilis tendon was harvested, folded into four strands, forming a cylindrical graft about 4 cm in length and 6 mm in diameter (Figure 3E). The graft was inserted into the hip joint cavity and secured (Figure 3F). The reconstructed labrum was checked for stability and anatomical restoration.

#### Treatment of the femoroacetabular impingement and wound closure

The traction was then released, and the knee and hip were flexed to explore the peripheral compartment of the hip joint. A cam deformity of the femoral head-neck junction was observed, causing impingement against the acetabulum during hip flexion. Under arthroscopic guidance, the femoral head-neck junction was reshaped (Figure 3G). After confirming the correct count of surgical instruments, the arthroscopic instruments were removed, and the incisions were sutured. The excised calcified labral tissue was sent for pathological examination (Figure 3H).

### Postoperative medication, imaging assessment, rehabilitation

#### Postoperative medication and imaging review

**Figure 4:**
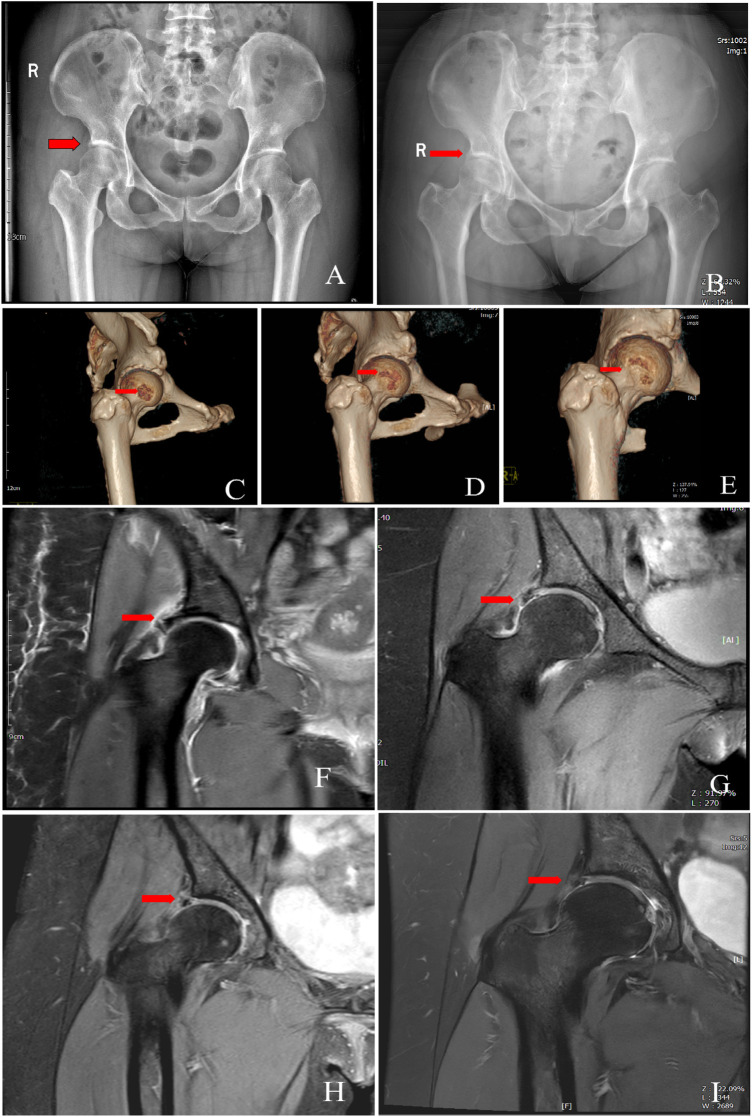
Postoperative radiographs [**(A)** immediate postoperative, **(B)** nine months postoperative, anteroposterior view] and the three-dimensional CT [**(C)** immediate postoperative, **(D)** six-month postoperative, **(E)** nine-month postoperative, reconstructed view] demonstrate resolution of the high-density opacity at the anterolateral margin of the right acetabulum and no obvious degenerative changes of the hip joint. **(F–I)** Postoperative MRI [**(F)** immediate postoperative, **(G)** three-month postoperative, **(H)** six-month postoperative, **(I)** nine-month postoperative, coronal view] show the repair of the labrum with an autologous gracilis tendon. The nine-month postoperative MRI revealed that the labral morphology had largely returned to normal. Neither appreciable joint effusion nor surrounding soft-tissue edema was observed.

Etoricoxib (60 mg, po, qd) was used for analgesia and prevention of heterotopic ossification and continued until the 4th week after surgery. Biqi Capsules (1.2 g, po, tid) were used for their anti-inflammatory effects and reduction of swelling for two weeks after surgery. Rivaroxaban (10 mg, po, qd) was used for anticoagulation to prevent lower extremity venous thrombosis within 4 weeks after surgery. Cefazolin sodium (2 g, iv) used for preventing infection within 24 h of the perioperative period. Postoperative x-ray and CT of the right hip showed resolution of the high-density area at the acetabular rim (Figures 4A-E). Postoperative MRI of the right hip revealed effusion and a reconstructed labrum in the right hip joint (Figure 4F).

#### Postoperative rehabilitation plan

Ankle pump exercises were started on the first day after surgery. On the postoperative first day, the patient was allowed to get out of bed for daily activities but with no weight-bearing on the affected limb. From postoperative day 3, passive range-of-motion exercises for the hip were initiated. After the postoperative 10th day, hip flexor stretching and thigh muscle strengthening exercises were added. From postoperative week 3, the patient was allowed to walk with crutches, bearing weight within a pain-free range and with full plantar contact on the ground. Passive hip movements were aimed at normalization to match the unaffected side by the postoperative third week, accompanied by hip drop and weight transfer exercises, including single-leg standing. As pain decreased and muscle function recovered, full weight-bearing was gradually achieved by 6 weeks, with early discontinuation of crutches and restoration of normal gait. Normal activities and sports such as jogging were gradually resumed between 3 and 6 months after surgery.

### Follow-Up

Follow-up plan after discharge: Follow-up evaluation was performed at 2 weeks, 1 month, 3 months, 6 months, and 9 months postoperatively. MRI of the right hip was performed at 3 months, 6 months, and 9 months postoperatively to observe postoperative changes (Figures 4G-I). At the nine-month follow-up, she had returned pain-free to normal activities and sports, including jogging. The visual analog scale (VAS) score decreased from a preoperative value of 7–1 at 3 months postoperatively, and further to 0 at both 6 and 9 months postoperatively. Similarly, the modified Harris Hip Score (mHHS) improved from a preoperative baseline of 60–95 at 3 months, reaching 98 at both 6 and 9 months postoperatively. The Non-Arthritic Hip Score (NAHS) showed a progressive increase from a preoperative score of 50–90 at 3 months, 96 at 6 months, and 97 at 9 months postoperatively. Consequently, patient satisfaction was rated as “very satisfied”.

## Discussion

FAI is a common hip joint disease, especially prevalent among young and active individuals. Its pathological mechanism involves abnormal contact between the femoral head–neck junction and the acetabular rim, leading to labral and cartilage damage, which in turn causes hip pain, limited mobility, and other symptoms (19). The labrum serves critical functions in the hip joint, including deepening the joint socket, increasing joint stability, and maintaining intra-articular fluid pressure. Advancements in imaging technology have recently drawn increasing attention to the diagnosis of labral calcification. Labral calcification may exacerbate hip joint pathology and compromise function. Moreover, it may synergize with impingement syndrome to create a self-perpetuating cycle. Existing literature has demonstrated a positive correlation between labral calcification volume and symptom severity (10, 20). However, systematic epidemiological data regarding large calcifications remain scarce, and the rarity of these cases hinders the accumulation of clinical experience. Further research is therefore warranted to enhance our understanding and management of this condition (20).

Evidence indicates that arthroscopic calcification debridement combined with labral repair and osteoplasty significantly alleviates symptoms in FAI patients with labral calcification (21, 22). Nevertheless, managing extensive labral calcifications presents specific surgical challenges; simple debridement may result in severe labral tissue defects, potentially compromising the labral “seal” effect and overall hip joint mechanical stability, and may even accelerate hip joint degeneration (13, 21). The critical intraoperative decision depends on a careful assessment of residual labral tissue quality and quantity to determine the optimal surgical strategy—debridement, repair, or reconstruction (22). If adequate healthy labral tissue remains (typically >3 mm in width), primary repair is recommended (8, 14, 18, 23). Conversely, severe labral atrophy or deficiency with insufficient residual tissue (<3 mm) warrants debridement or labral reconstruction (18, 22–24).

Existing literature suggests that arthroscopic management strategies for FAI complicated by concomitant labral calcification have primarily consisted of labral debridement or repair (14, 16, 18), yet there is a paucity of literature concerning labral reconstruction—likely attributable to a relatively small calcification burden in reported cases. The reported patient was a young sports enthusiast. Upon thorough removal of the calcific deposits, intraoperative findings revealed that the remaining labrum exhibited compromised integrity, presenting with a substantial defect that was not amenable to suture repair. Additionally, preoperative risk factors for hip instability were present, including an LCE angle of 28°. Performing acetabular labral debridement alone would compromise postoperative hip stability and accelerate joint degeneration. To restore biomechanical function and delay the progression of osteoarthritis, we performed arthroscopic femoral cam osteoplasty, debridement of the calcifications and labral reconstruction with autologous gracilis tendon. This approach effectively eliminates impingement factors, restore labral continuity, and maintains hip joint stability, thereby optimizing long-term prognosis (20, 21), especially in young patients at high risk. Intraoperatively, we implemented the “three-needless” concept, namely no perineal post, no fluoroscopy, and no urinary catheterization. Instead, the trendelenburg position combined with an abdominal binder was employed to provide counter-traction to the bilateral lower limb traction, thereby enlarging the hip joint space. This approach eliminates the risk of pudendal nerve injury associated with the perineal post, reduces radiation exposure to both patients and the surgical team, and minimizes the risk of catheterization-related complications (25, 26).

Current research identifies several graft options for acetabular labral reconstruction, including semitendinosus tendon, iliotibial band, peroneus longus tendon, autologous joint capsule, and fresh meniscus allografts (27, 28). Recent evidence demonstrates that fresh meniscus allografts outperform fresh tendon allografts in cell viability, extracellular matrix, geometric morphology, material properties, and early functional recovery, suggesting potential advantages in specific clinical scenarios (28). However, systematic reviews show comparable efficacy between autologous and allogeneic grafts, with selection based on a comprehensive assessment of the patient's condition, surgical trauma, and the surgeon's experience (29). In this case, the autologous gracilis tendon was selected based on the following rationale: (1) Biological safety: This approach eliminates the risk of disease transmission and immune rejection inherent to allografts (30); (2) Biomechanical compatibility: The tensile properties of gracilis tendon are similar to those of the native labrum, providing near-normal mechanical performance (31); (3) Minimally invasive advantage: The harvest incision is smaller than that required for the iliotibial band. Furthermore, the gracilis tendon dimensions closely match the labral defect, thereby reducing excessive tissue resection compared with that of the semitendinosus tendon; (4) Capsular preservation: Given the patient's LCE angle of 28°(approaching the lower limit of normal), local capsular harvest would compromise capsular integrity, which contradicts the intraoperative strategy of capsular repair and reinforcement. In conclusion, the gracilis tendon graft may be preferable in this specific clinical context for balancing biomechanical reconstruction with joint stability in this specific clinical context.

Although many studies on postoperative outcomes following hip labral reconstruction require a minimum 2-year follow-up (32, 33), the literature also documents shorter follow-up periods, including case series that report outcomes at 6 months (30). Scanaliato JP reported that 86.7% returned to sport at a median of 6.6 (range 3–9) months postoperatively (34), suggesting that meaningful clinical recovery may occur within the first year. While the present case demonstrated promising short-term improvement, long-term follow-up is required to assess graft durability, osteoarthritis progression, and late failure risk. This case report demonstrates that arthroscopic calcified lesion debridement with concomitant labral reconstruction is a viable therapeutic strategy for patients with FAI and extensive labral calcifications. This surgical approach may facilitate symptom alleviation, restoration of hip joint function, return to sporting activities, and an improvement in the patient's quality of life.

## Data Availability

The original contributions presented in the study are included in the article/Supplementary Material, further inquiries can be directed to the corresponding authors.
